# Ethnic disparities of poor households by a multilevel analysis of household and contextual effects: Evidence from a multi-ethnic county of China

**DOI:** 10.1371/journal.pone.0313533

**Published:** 2024-12-12

**Authors:** Junfeng Tian, Binyan Wang, Li Ma, Yunyan Li, Shijun Wang

**Affiliations:** 1 School of Public Policy and Administration, Chongqing University, Chongqing, China; 2 School of Architecture and Urban Planning, Chongqing University, Chongqing, China; 3 School of Public Administration, Xi’an University of Architecture and Technology, Xi’an, China; 4 Key Laboratory of Monitoring, Evaluation and Early Warning of Territorial Spatial Planning Implementation, Ministry of Natural Resources, Chongqing, China; 5 School of Architecture, State Key Lab of Subtropical Building Science, South China University of Technology, Guangzhou, China; 6 School of Geographical Sciences, Northeast Normal University, Changchun, China; Sam Houston State University, UNITED STATES OF AMERICA

## Abstract

Poverty in multi-ethnic regions has always been a concern due to its complex factors and persistent nature. Using a sample of 8,482 ethnic majority-headed households and 2,011 ethnic minority-headed households distributed in 200 villages of Wangqing County, China, this study uses hierarchical linear models to examine the factors of income at the household level, the ethnic disparities of the household-level effect, and the contextual effect on household-level outcomes. The findings suggest that, in comparison to the majority group, there exists a smaller income gap between male-headed and female-headed poor households within the minority group. Moreover, the positive impact of participating in off-farm work and receiving welfare payments on the income of poor households is significantly stronger within the minority group. These results not only highlight ethnic disparities in household-level effects but also underscore potential influences of ethnicity on the income dynamics of poor households. The contextual effect demonstrates that modifying the environment of poor households can either enhance or diminish some of the impacts resulting from factors at the household level, thereby facilitating the formulation of more effective targeting strategies at different levels. This study provides an important reference for understanding the ethnic differences of poor households and the mechanism of their income from a multilevel perspective.

## Introduction

Eradicating extreme poverty for all people everywhere by 2030 is a pivotal goal of the 2030 Agenda for Sustainable Development. Globally, although steady progress has been made in poverty reduction for 25 consecutive years before the COVID-19 pandemic, the pace of poverty alleviation is slowing down. The Sustainable Development Goals Report 2022 revealed that more than three years of poverty reduction achievements have been erased from 2019 to 2020, with the global poverty rate increasing sharply from 8.3% to 9.2% [1], indicating that more people are trapped in the plight of insufficient livelihood resilience, lack of opportunities, and substandard quality of their living environment accompanied by poverty. It also poses a greater challenge to the United Nations Sustainable Development Goal of ending poverty by 2030.

Compared with the general public, the livelihood resilience of the poor is more fragile and vulnerable to economic, material, and structural damage [2]. Furthermore, in multi-ethnic regions, the problem of poverty is more complicated and needs to be addressed strategically [3, 4]. In a multi-ethnic region, languages, lifestyles, and cultural heritage are often different among different ethnic groups. Some high-efficiency poverty reduction strategies for an ethnic group are low-inefficiency or even ineffective for another ethnic group. Therefore, in areas where multi-ethnic settlements and poverty problems overlap, the formulation of poverty reduction strategies needs to consider not only the effectiveness of poverty reduction projects themselves but also the differences and specific needs of different ethnic groups. Many empirical studies have revealed that within a country or region, ethnic minorities often have a higher incidence of poverty than the ethnic majority [5], a conclusion drawn from the following two aspects. One aspect is the fact that the incidence of poverty in multi-ethnic regions is higher than that in regions mostly composed of major ethnic groups. The other aspect is the fact that the proportion of the poor in ethnic minorities is higher than that in the ethnic majority. Therefore, regions where the ethnic minorities are poorer than the ethnic majority are often the focus of research [6, 7], and little attention has been paid to regions where the incidence of poverty among ethnic minorities is lower than in the ethnic majority.

Many studies have investigated the factors of household poverty, showing that poverty is associated with factors at different levels, including factors at the levels of household and context [6]. Specifically, the impact of ethnicity on poverty has already been confirmed in existing research. A study on China by Gustafsson and Ding [8] concluded that the differences between ethnic minorities and ethnic majorities in physiological characteristics, education level, family size, cultural habits, and access to and utilization of opportunities lead to a higher incidence of poverty among minority groups. Research in agricultural areas in Vietnam also showed that language barriers and cultural differences between different ethnic groups have an important impact on rural poverty, even greater than the effects of natural isolation [5, 8, 9]. Research has also pointed out that ethnic heterogeneity can affect various economic and entrepreneurship activities [4, 6, 10].

More comprehensively, capital at the household level, including natural, social, economic, political, and human capital, is proven to be essential to poverty status. Households headed by females, elders, ethnic minorities, indigenous people, people in low castes, people with low education levels, people without labor capacity, and households with sick family members, educational burden, and high dependency ratio are shown to be more vulnerable to poverty [5, 8, 11–21]. Among these groups, health shocks are revealed to be the primary factor of poverty at the household level, affecting household poverty status in multiple ways. For example, unemployment caused by health problems directly reduces family income. Medical or funeral expenditure increases the family burden, and more time and resources are needed for health care [22, 23]. In Kenya, Uganda, India, and Peru, poor health and high medical costs are also found to be the leading factors of household poverty. Krishna [24] also pointed out that the interaction between health and debt is one of the important reasons for Indian households falling into poverty.

Family size, welfare payments, and participation in non-agricultural activities are also important variables affecting household poverty. However, the influence mechanism and degree of these variables on poverty are varied in different study cases. Research conducted in Zimbabwe’s semi-arid regions showed that large family size is an important factor of poverty among families [12]. Analyses of Korean households with disabled members found that the larger the family size, the less likely it is to fall into poverty because more family members can share the burden [17, 18]. The impact of welfare payments on poverty is inconsistent among different groups [25]. Sometimes, welfare payments alleviate poverty by directly increasing household income, but sometimes it weakens the independent initiative of households, thereby reducing household income. The impact on poverty of engaging in non-agricultural activities varies with geographical location, social system, and stage of development. Consequently, to take accurate and efficient poverty alleviation actions, it is necessary to research different types of regions to find the factors of local poverty.

At the environmental level, significant relationships were also observed between household poverty and the context in which people lived [13, 26, 27]. For rural households, the natural capital of the context is considered to be important and can have a lasting effect by influencing other dimensions of capital, such as economic and social aspects [28]. In most agricultural areas, agricultural production potential directly affects the type of economic activity and income of farmers. As a consequence, when agricultural growth is the key determinant of rural poverty reduction, topography, soil, rainfall, and other natural geographical factors are closely related to farmers’ income [29, 30]. As an important supplementary means when natural geographical capital is insufficient, irrigation facilities are also closely related to the income of farmers [30]. Climate can also indirectly regulate income by acting on human health and agricultural productivity, thereby affecting poverty [22, 31, 32].

The accessibility of traffic, economics, and markets is crucial for rural households if they are to escape poverty [30, 33–36]. The distance to neighboring urban areas affects the purchase of production goods and the sale of agricultural products for farmers. The farther the distance to the urban areas, the fewer production goods and services are obtained by farmers at reasonable cost. Unit prices are higher and goods are of lower quality. Simultaneously, the unit price of agricultural products is lower, the sale choices are fewer, and transportation costs are higher. In addition, the long distance may also hinder government officials and professional and technical personnel from visiting remote areas, thereby increasing the possibility of political neglect in these areas [37]. Empirical studies on rural areas in the United States and semi-arid areas in Zimbabwe have pointed out that there is a significant correlation between geographical remoteness and the high incidence of long-term poverty. As the distance to metropolitan areas increases, the incidence of poverty gradually increases in response to the attenuation of urban radiation effects, imperfect commuting, and lower demand for rural labor [12, 38]. Conversely, a fast and convenient connection to a transportation infrastructure weakens the adverse effects of remoteness to a certain extent. Improving the accessibility of traffic facilities provides support for rapid material and energy exchange, as well as for the exchange and diffusion of new technologies [12], thereby weakening the development lag caused by geographical barriers. A study conducted in Mozambique showed that denser road networks and better transport facilities can increase the adoption of new agricultural technologies by improving market accessibility and compensating for the geographical disadvantages of marginalization [39]. In Tanzania, improved road conditions also had a positive effect on families engaging in new or more diverse employment activities [40].

Local socioeconomic status also affects poverty [20, 41]. The backwardness and imbalance of economic development have been proven to be one of the reasons for the lack of ability to abolish poverty in the western ethnic areas of China [42]. In northeast China, famous as a grain production base, the amount of cultivated land is the important material basis for agriculture [19]. Adequate human resources are fundamental to ensuring rural development and maintaining rural vitality. The serious dilemma of population loss faced by Yanbian Korean Autonomous Prefecture in northeast China leads to the aggravation of the aging problem, the prominent hollowing out of rural areas, and rural underdevelopment problems [19].

Based on the above, we propose that the following aspects need more attention and further analysis. (1) The research solely focusing on poor households is limited, and there is a lack of studies directly comparing poor households among ethnic minorities and majorities in multi-ethnic regions. Previous studies have predominantly taken all the residents in the study area as the object of investigation to compare and analyze disparities between ethnic minorities and ethnic majorities and the factors of poverty [8, 43]. For example, a research conducted by Gustafsson and Ding (2009) [8] investigated the poverty among ethnic minorities and the majority in rural China, using a large sample covering 22 provinces. And another research conducted by Gradín (2015) [43] investigated the nature of the differential in poverty by ethnicity in rural China. Data for both above two studies is provided by the rural household survey for 2002 collected by the China Household Income Project (CHIP). The CHIP 2002 comprises three components: urban household survey, rural household survey, and floating population survey, aiming to ensure comprehensive representation of all households in China. Consequently, they investigated the disparities between ethnic minorities and ethnic majorities in the overall sample within the designated research area. (2) Previous studies have investigated the factors of poverty by taking ethnicity as one of the factors [13, 44, 45]. However, there is insufficient research focused on the disparate impact of poverty factors across different ethnic groups. We propose that conducting a comprehensive examination of the impact of disparities in poverty determinants among diverse ethnic groups will facilitate a more nuanced understanding of the intricate relationship between ethnicity and poverty. (3) Lots of studies have chosen case areas in multi-ethnic regions where ethnic minorities have a higher incidence of poverty than the ethnic majority in China [6, 7]. The attention given to multi-ethnic regions where the poverty rate among ethnic minorities is lower than that of the ethnic majority is insufficient. Furthermore, in certain multi-ethnic regions, the implementation of an extensive array of poverty reduction policies specifically targeting ethnic minorities has resulted in a comparatively lower incidence of poverty among these minority groups compared to the ethnic majority. The neglect of these regions and the persistent long-term implementation of targeted poverty reduction measures that primarily benefit ethnic minorities may give rise to emerging disparities. (4) Previous studies have confirmed that household income is influenced by both household characteristics and environmental features. However, the hierarchical structure has been overlooked in many studies, and single-level methods have been relied upon due to limitations in data availability and methodological constraints, as highlighted by previous research conducted by Kim et al. (2016) [15] and Wang et al. (2019) [26]. This approach may result in biased parameter estimates, incorrect test results, and flawed conclusions regarding effect sizes [14, 46]. Consequently, the existing research fails to meet the criteria necessary for informing policies aimed at eradicating poverty through multi-level approaches and interventions across various governmental tiers.

Therefore, this paper aims to fill the noted research gaps by using a multilevel approach to explore the ethnic disparities of poor households. Taking Wangqing County as a case study, where the poverty rate of ethnic minorities is comparatively lower than that of the ethnic majority, we investigate the impact of the household-level variables and their ethnic disparities on outcomes, while also exploring the contextual effect. The remainder of this paper is as follows. The materials and methods section shows the study area, data, and research methods. The results section provides the finding of the analysis conducted. The discussion and the conclusion section encompasses a comprehensive dscourse on the outcomes obtained.

## Materials and methods

### Study area

Wangqing County, a representative county of Yanbian Korean Autonomous Prefecture, faces serious problems of population outflow, a declining economy, and ongoing urban shrinkage [47]. It is located in Jilin Province, Northeast China, 40 kilometers from Russia and 18 kilometers from North Korea. With a total area of 9,016 km^2^, Wangqing County is divided into nine townships and 200 administrative villages. It is composed of 25 recognized ethnic groups, including the Han, the Korean, and the Manchu groups, with varying languages, lifestyles, and cultural heritage, 24 of which are classified as ethnic minorities. The Han accounted for 68.39% of the total population at the end of 2015. The next largest ethnic groups are the Koreans, accounting for 26.42%, the Manchu, accounting for 4.92%, and others accounting for 0.27%. In this paper, the poor households are divided into the majority group, namely the Han, and the minority group, namely the other 24 ethnic minorities. It is worth noting that the poverty rate of the minority group in Wangqing County is only 45% of that of the majority group.

### Data

Although the poor households not only face income insufficiency but also encounter limitations on other rights, increasing household incomes can significantly enhance livelihood resilience [20, 45]. Therefore, we consider annual per capita household income (referred to as "income" hereafter) as the dependent variable for analyzing its influencing factors and exploring the ethnic disparities in their impacts. The dependent variables at the household and environmental levels are shown in Table 1.

**Table 1:** Variables collected in this study.

Variables	Definitions
Dependent variable	
Income	Annual per capita household income
Independent variables	
Household characteristics	
Age	Age of the household head (years)
Gender	Gender of the household head (male = 0, female = 1)
Ethnicity	Ethnicity of the household head (majority = 0, minority = 1)
Education	Education level of the household head (illiterate or semi-illiterate, primary school = 2, middle school = 3, high school = 4, college or above = 5)
Disease	Disease status of household head (none = 0, chronic disease = 1, serious disease = 2)
Disability	Disability status of household head (none = 0, disabled = 1)
Labor capacity	Labor capacity of household head (no ability = 0, normal ability = 1)
Family size	The number of people living in a household together (person)
Dependency ratio	Ratio of dependent individuals (younger than16 years or older than 60 years) per family (%)
Student	Number of students in school (none = 0, one or more students = 1)
Off-farm work	Location of off-farm work for family members (none = 0, within the township = 1, outside the township = 2)
Welfare payments	Financial assistance from government (none = 0, dibao [welfare payment covering basic living expenses] or wubao (welfare payment covering food, clothing, healthcare, shelter, and funeral expenses = 1)
Environment features	
Altitude	Average elevation of village (m)
Terrain	Elevation range from the lowest point in village to the highest (m)
Slope	Average slope of village (°)
Slope change	Difference between maximum and minimum slope in village (°)
Rainfall	Average annual rainfall in village (mm)
Temperature	Average annual temperature in village (°C)
Distance to nearest county-level road	Distance of village to the nearest county-level road (km)
Distance to nearest township-level road	Distance of village to the nearest township-level road (km)
Distance to township center	Distance from village to the nearest township center (km)
Travel time to county center	The travel time from village to the nearest county center, as reported by Baidu Map (minute)
Distance to nearest river	Distance from village to the nearest main river (km)
Village size	Total population of village (persons)
Poverty size	The poor of village (persons)
Poverty rate	Ratio of poor in village (%)
Ratio of minority	Ratio of minority in village (%)
Hollowing	Ratio of nonpermanent residents in village (%)
Cultivated land	Landholding size per capita in village (mu~0.067 ha)

The data set at the household level was collected from a survey conducted by the Office of Poverty Alleviation and Development (OPAD) of Wangqing County, containing the basic information of 10,493 poor households scattered in 200 villages. The criterion for identifying poor households is defined as the failure to meet the requirements of “One income, Two concerns, or Three assurances” published in the Outline of China’s Rural Poverty Alleviation and Development (2011–2020) issued by the State Council of China (https://www.gov.cn/gongbao/content/2011/content_2020905.htm). Specifically, the information on the age, gender, ethnicity, education, health status, labor capacity of the household head and the family size, dependency ratio, education burden, welfare payments, and participation in off-farm work of the household were selected as independent variables at the household level, in line with existing studies and taking data accessibility into consideration.

Village features were regarded as proxies of the context at the environmental level in this paper for the following two reasons. Village features are the important environment supporting the villagers’ production and living [27, 48, 49]. As the core unit of rural management in China, administrative villages implement self-governance of villagers, villagers building houses and related living facilities, and sharing the resources and facilities within the village. However, the number of villages is too small to meet the requirements of taking its superior administrative unit as another higher environment level when using the hierarchical linear models in this study. The socioeconomic information of the villages was also derived from the survey conducted by OPAD, and the physical geography of the villages was obtained from the Resources and Environmental Science and Data Center of the Chinese Academy of Sciences (http://www.resdc.cn). The accessibility of the villages to transportation and facilities was calculated based on the regional basic geographic dataset, the 1:250,000 database of the National Geomatics Center of China (http://www.webmap.cn/commres.do?method=result25W), and Baidu Maps (https://map.baidu.com/). The necessary registration, correction, clipping, coordinate transformation, and other processing of the data used in this study were performed.

## Methods

### Hierarchical linear models

First, we run the null model derived from the hierarchical linear models to confirm whether it is necessary to apply the hierarchical linear models in this study based on the value of the intraclass correlation coefficient (ICC) [46]. The formulas for the null model are as follows:

$$Y_{ij}=\beta_{0j}+\varepsilon_{ij}\;Variance(\varepsilon_{ij})=\delta^{2}$$ (1)

$$\beta_{0j}=\gamma_{00}+\mu_{0j}\;Variance(\mu_{0j})=\tau_{00}$$ (2)

ICC=τ00τ00+δ2 (3)

where,*Y*_*ij*_ is the annual per capita household income of poor household *i* living in village *j*; *β*_0*j*_ is the mean outcome for the village *j*, and *γ*_00_ is the grand mean outcome for all poor households. We assumed that *ε*_*ij*_ ~ *N*(0,*δ*^2^) for *i* = 1,2,…,*n* poor households in village *j*, *j* = 1,2,…,200 villages, and *μ*_0*j*_ ~ *N*(0,*τ*_00_), a random error in village level. *δ*^2^ and *τ*_00_ denote the variance at the household level and village level, respectively.

Then, a random-coefficient regression model derived from the hierarchical linear models was applied to examine the effect of variables at the household level without the contextual effect and to identify the household-level variables that have significantly varied relations with income among villages. The formulas of the random-coefficient regression model are as follows:

$$Y_{ij}=\beta_{0j}+\beta_{1j}X_{ij}+\varepsilon_{ij}$$ (4)

$$\beta_{0j}=\gamma_{00}+\mu_{0j}$$ (5)

$$\beta_{1j}=\gamma_{10}+\mu_{1j}$$ (6)

where,*X*_*ij*_ is the predictor of household *i* in village *j*. *β*_0*j*_ and *β*_1*j*_ are the intercept and slope, respectively. *γ*_00_ and *γ*_10_ are the mean values of *β*_0*j*_ and *β*_1*j*_, respectively, and *μ*_0*j*_ and *μ*_1*j*_ are the random elements of *β*_0*j*_ and *β*_1*j*_, respectively, representing the variation among the villages.

Finally, the full model derived from the hierarchical linear models was applied to explore how variables at the village level influence the effect on income of variables at the household level, namely the contextual effect. The full model is expressed as follows:

$$Y_{ij}=\beta_{0j}+\beta_{1j}X_{ij}+\varepsilon_{ij}$$ (7)

$$\beta_{0j}=\gamma_{00}+\gamma_{01}W_{j}+\mu_{0j}$$ (8)

$$\beta_{1j}=\gamma_{10}+\gamma_{11}W_{j}+\mu_{1j}$$ (9)

where, *W*_*j*_ is the predictor at the environment level and *γ*_01_ and *γ*_11_ are the slope of the equations at the environment level.

## Results

### The household-level effect

In this section, the coefficient of variation test and multicollinearity diagnosis were applied to screen the variables before running the null model. The results of the null model (see S1 Table) indicated that 95.00% of the expected income of poor households in Wangqing County ranged from 4,639.99 CNY to 7,159.77 CNY. The results also show that the fixed effect and random effect passed the significance test (p < 0.001), and the ICC was 12.02%, confirming the rationality and necessity of applying hierarchical linear models.

The variables with a coefficient of variation less than 15.00% and those exhibiting multicollinearity were eliminated before conducting the random effects regression models (refer to S2 Table). Ten variables significantly associated with the income of poor households in the univariate random effect regression model (S3 Table) were put into the multivariate random effect model. Table 2 shows the results from the multivariate random effect model for the household-level effect for poor households in Wangqing County. Apart from the age, education level, and disability status of household heads, the other seven variables show statistically significant relationships with income of poor households for the total sample. In this study, due to the aging trend of the sample population (refer to S4 Table) and a low variation of age among respondents, there was a lack of statistically significant correlation between age and income. Similarly, the educational attainment of the sampled population in this study tends to be comparatively lower, predominantly concentrated at primary school levels or lower. Consequently, there does not exist a significant correlation between educational level and income. The observation that households with disabled family members do not necessarily have a low income may be attributed to government-provided assistance.

**Table 2:** Results of the multivariate random effects regression model.

	Regression coefficient			Regression coefficient variance		
	Parameter	Coefficient	Standard error	parameter	standard error	variance component
	G_00_	5 900.18***	99.03	U_0_	1 285.26***	1 651 905
Age	G_10_	5.39	4.72	U_1_	26.57	706
Gender	G_20_	−542.43***	85.27	U_2_	513.52	263 701
Education	G_30_	56.32	47.94	U_3_	135.73	18 422
Disease	G_40_	−423.44***	99.65	U_4_	609.84	371 905
Disability	G_50_	−216.17	117.54	U_5_	726.11	527 233
Labor capacity	G_60_	1022.41***	130.15	U_6_	1 034.82	1 070 861
Family size	G_70_	−788.30***	68.14	U_7_	518.68***	269 032
Dependency ratio	G_80_	−592.64***	123.25	U_8_	881.61**	777 233
Off-farm work	G_90_	1 408.30***	185.13	U_9_	1 484.10***	2 202 558
Welfare payments	G_100_	469.18***	111.55	U_10_	913.75***	834 942
				R	3 065.09	9 394 772

* p <0.1,

** p <0.05, and

*** p<0.01

Consistent with the results of the univariate random effect regression model, the results of the multivariate random effect model show that the gender and disease status of household head, family size, and dependency ratio of household are significantly negatively correlated with the income of poor households, and the labor capacity of household head and off-farm work of the household are significantly positively correlated with the income of poor households.

Compared with male-headed households, the income of female-headed households is 542.43 CNY lower on average, indicating that there is still a significant gender gap in the income of poor households in Wangqing County. The 95% CI of the coefficient ranges from −590.50 to −490.36, meaning that the income of female-headed households is lower than that of male-headed households in all villages after the other variables are controlled, and the coefficients of gender with income are consistently negative, with no significant variation among villages (P > 0.100). As expected, poor households headed by a person with labor capacity earn more than poor households headed by a person without labor capacity. Surprisingly, the income gap between the above two groups is as high as 1022.41 CNY on average. The 95% CI of the coefficient of labor capacity ranges from −400.76 to 2445.58, showing large disparities in the effect of labor capacity on income among villages, but it is not statistically significant (P > 0.500). The coefficient of family size with income of the poor households is negative on average; with one person increase, the income decreases by 788.30 CNY. However, the 95% CI of the coefficient ranges from −3307.42 to 1730.82 with statistically significant variation among villages (P<0.001), demonstrating that there are notable differences in the effect of family size on poor households’ income. The effect varies significantly in both direction and degree among villages, showing that in some villages, the reduction of family size increases the income of poor families, while in others, the expansion of family size increases the income of poor households. Moreover, in some villages, family size has little effect on the income of poor households. This finding suggests that there is spatial heterogeneity in the effect of family size on household income. With the increase in the dependency ratio, the income of poor households decreases. There are also significant disparities in the effect of the dependency ratio on the income of poor households among villages (P < 0.05), with the 95% CI ranging from −1599.14 to 413.85. Participating in off-farm work increases the income of poor households significantly, with the coefficient of off-farm work as high as 1408.30, the highest for all the coefficients of variables at the household level, demonstrating that poor households with members engaged in off-farm work earn 1408.30 CNY on average more than poor households with no member engaged in off-farm work. There are significant variations in the coefficient of off-farm work on the income of poor households among villages, with the 95% CI ranging from −615.95 to 3436.56, indicating that the effect of participating in off-farm work on income varies among villages, with spatial heterogeneity. However, it is inconsistent that the coefficient of welfare payments with income changes from negative in the results of the univariate random effect regression model to positive in the results of the multivariate random effect regression model, demonstrating that assistance from the government increases the income of poor households significantly after the other variables at the household level are controlled. Specifically, compared to those who do not receive government assistance, the income of poor households with government assistance increases by 469.18 CNY on average in Wangqing County. However, the 95% CI of the coefficient ranges from −2439.66 to 3378.01, meaning the effect of welfare payments on income varies significantly among different villages (P < 0.05).

### Ethnic disparities in the household-level effect

In order to examine the disparity of the household-level effect between the majority group and minority group, we put the interaction of ethnicity with other household characters into the random-coefficient regression model. Again, we performed a coefficient of variation test and multicollinearity diagnosis to screen variables that can be put into the household level in a random-coefficient regression model (see results in S5 Table). As Table 3 shows, the coefficients of ethnicity*gender and ethnicity*off-farm work with income are statistically significant at the 0.05 level, and the coefficient of ethnicity*welfare payments with income is statistically significant at the 0.1 level, revealing that there are significant ethnic disparities in the effect of the above three variables on the income of poor households in Wangqing County.

**Table 3:** Results of the multivariate random effects regression model with the interaction term.

Interaction term	Parameter	Coefficient	Standard error
Ethnicity*Gender	G_110_	463.58***	170.76
Ethnicity*Disease	G_120_	33.74	171.75
Ethnicity*Disability	G_130_	−112.91	195.84
Ethnicity*Labor capacity	G_140_	−341.22	276.55
Ethnicity*Family size	G_150_	−147.69	109.23
Ethnicity*Off-farm work	G_160_	1 603.56***	464.32
Ethnicity*Welfare payments	G_170_	306.42*	156.54

For simplicity, the estimation of the slopes for the other household-level variables is not listed here.

* p <0.1,

** p <0.05, and

*** p<0.01

As the majority group of poor households is coded as 0, and the minority group of poor households is coded as 1 in this study, the coefficient of the interaction term is the disparity of the household-level effect on income between the minority group and the majority group. Because the coefficient of gender with income is negative, the positive effect of the interaction of ethnicity*gender on income indicates that the negative effect of gender on income is significantly weaker in the minority group than in the majority group. This result demonstrates that the income gap between male-headed and female-headed poor households in the majority group is significantly bigger than that in the minority group. This also verifies that there are notable ethnic disparities in the effect of gender on income for poor households in Wangqing County.

Both the coefficient of off-farm work with income and the coefficient of ethnicity*off-work with income are positive, demonstrating that the positive effect of participating in off-farm work on income is significantly stronger for the minority group than for the majority group. Compared with poor households in the majority group, the income gap between households with one or more family members participating in off-farm work and households with no family members participating in off-farm work is significantly bigger in the minority group. This result not only indicates that participation in off-farm work increases the income of poor households but also further reveals that the anti-poverty effect of providing off-farm employment opportunities to poor households of different ethnic groups is different.

The stronger positive effect of welfare payments on the income of poor households is evident in the minority group rather than in the majority group. The significant disparity in the effect of welfare payments on income between the majority group and the minority group demonstrates that ethnicity does influence the poverty reduction effect of government assistance. Therefore, more refined government assistance should be designed for different ethnic groups.

### Results of the contextual effect

The variables that significantly impact the income of poor households at the household level and the variables with a coefficient of variation greater than 15.00% at the environment level are incorporated into the full model. Table 4 shows the results of the full model.

**Table 4:** Results of the full model.

	Parameter	Coefficient	Standard error
Gender	G_10_	−725.82***	102.30
Disease	G_20_	−369.96***	67.66
Labor capacity	G_30_	1 097.49***	167.70
Family size	G_40_	−837.66***	87.35
	G_415 (family size* hollowing)_	−620.30**	309.89
Dependency ratio	G_50_	−374.00***	118.54
	G_53 (dependency ratio*slope)_	112.84**	52.55
	G_511 (dependency ratio*village size)_	0.93**	0.44
	G_514 (dependency ratio*ratio of minority)_	1 331.57***	456.06
Off-farm work	G_60_	1 316.55***	209.05
	G_61 (off-farm work*altitude)_	−5.48***	1.70
	G_63 (off-farm work*;slope)_	216.61***	77.69
	G_64 (off-farm work*slope changes)_	-77.91**	34.31
	G_610 (off-farm work*distance to nearest river)_	94.62**	37.92
Welfare payments	G_70_	511.44***	128.61
	G_77 (welfare payments*distance to nearest township-level road)_	54.61**	26.14
Ethnicity*Gender	G_80_	556.19***	178.05
Ethnicity*Off-farm work	G_90_	1 185.06***	456.45
Ethnicity*Welfare payments	G_100_	−106.21	121.67

For simplicity, variables at the environmental level that did not pass the t-test are not listed here.

* p <0.1,

** p <0.05, and

*** p<0.01

The degree of hollowing out of villages at the environmental level significantly affects the effect of family size on income (P < 0.05). Specifically, the degree of hollowing out of villages has a negative impact on the slope of family size, consistent with the direction of the impact of family size on the income of poor households, indicating that the degree of hollowing out of villages enhances the negative impact of family size on the income of poor households. Therefore, when other conditions remain constant, the increase in family size in villages with a higher hollowing ratio has a stronger effect on the reduction of income of poor households than in villages with a lower hollowing ratio.

The average slope, village size, and ratio of ethnic minorities at the environmental level have an impact on the relationship between the dependency ratio and income of poor households at the 95.00% significance level, and these three variables are positively correlated with the slope of dependency ratio, which is opposite to the impact of dependency ratio on the income of poor households, demonstrating that the increase of average slope, village size, and the ratio of ethnic minorities weaken the negative impact of dependency ratio on income.

The relationship between off-farm work and the income of poor households is significantly affected by the variables of altitude, average slope, slope change, and distance to the nearest river at the environmental level (P < 0.05). The impact of village altitude and slope changes on the slope of off-farm work is negative, which is contrary to the positive impact of off-farm work on the income of poor households. This indicates that village altitude and slope changes weaken the effect of participation in off-farm work on the income of poor households. Conversely, the average slope of the village and the distance from the village to the nearest river have a positive impact on the slope of off-farm work, revealing that the increase in the average slope of the village and the increase in the distance from the village to the nearest river enhance the effect of participation in off-farm work on the income of poor households.

The influence of the distance to the nearest township-level road on the relationship between welfare payments and income of poor households passed the 95.00% significance level test. The distance to the nearest township-level road has a positive impact on the slope of welfare payments, consistent with the positive effect of welfare payments on the incomes of poor households. That is, with the increase in the distance to the nearest township-level road, the positive impact of welfare payments on the income of poor households is stronger.

## Discussion and conclusion

### Significant disparities identified between the majority and minority groups

This paper examined the ethnic differences of poor households in a specific type of area where the poverty rate of the ethnic minorities is lower than that of the ethnic majority. The characteristics of the poor households between the minority groups and the majority group showed significant differences within the study area (refer to S4 Table). Compared with the poor households in the majority group, the aging situation of the household heads in the minority group was more evident, and a higher percentage of households are headed by females, sick people, and people without labor capacity in the minority group than in the majority group, although ageing, sickness and incapacity are prominent in both groups. In addition, poor households in minority groups have smaller family sizes, larger dependency ratios, and more reliance on welfare payments. Conversely, the heads of poor households in the majority group have lower education levels and a higher proportion of disabilities. These results are not exactly the same as the differences between the ethnic minorities and the ethnic majority found in the regions where the poverty incidence of ethnic minorities is higher than that of the ethnic majority, and the results are also different from those for when all the population in the region is taken as the research object; some conclusions are even opposite. These results are conducive to our broader understanding of the differences between the ethnic minorities and the ethnic majority within poor households.

### Ethnic disparities exist in the impact of household-level factors on income of poor households

We not only examine the role of ethnicity as a direct factor affecting the income of poor households, but we also investigate ethnic disparities in the impact of other household-level factors to identify potential influences on the income of these households. In this paper, when only ethnicity and other characteristic variables at the household level are taken as independent variables to examine their effect on the income of poor households, ethnicity does not show a significant impact on the income of poor households, consistent with the research published by Li and Ding [50] and Gustafsson and Ding [51]. However, when we put the interactive term of ethnicity and other characters into the random-coefficient regression model, there are significant ethnic differences in the impact of gender, off-farm work, and welfare payments on the income of poor households. That is to say, although there is no direct significant correlation between ethnicity and the income of poor households in the case area, ethnicity indirectly impacts the income of poor households by reacting differently to the effect of gender, off-farm work, and welfare payments on the income of poor households.

The differential impact of gender on income may be attributed to the distinct family demographic composition and age structure observed among ethnic minorities compared to the ethnic majority. Regarding family structure, ethnic minorities in our study, predominantly composed of ethnic Korean, exhibit distinctive patterns. The ethnic Korean families demonstrate a higher overall level of aging and are more likely to comprise nuclear families consisting of old married couples or single-person households, thereby attenuating the influence of gender disparities on income inequalities. Compared to ethnic Korean households, Han households exhibit a tendency towards larger family units characterized by the cohabitation of multiple generations (the average household sizes for the majority and minority groups are 1.72 and 1.53, respectively, with statistically significant differences at p≤0.001). This is particularly evident in terms of sons residing together with their parents, given their predominant role as primary breadwinners within these families, thereby exacerbating the gender income gap. Furthermore, the ethnic Han group experiences a lower overall level of aging, which contributes to income disparities.

The differential impact of participating in off-farm work on income is related to the different work place and wage derived from such work between the two ethnic groups. As demonstrated by Wang and Tian (2015) [52], Wang et al. (2017) [19], and Li (2018) [53], a significant proportion of ethnic Korean households opt to work in South Korea, which boasts stronger economic development and higher wage income, while Han households predominantly engage in non-agricultural activities within China. The wage disparity between China and South Korea accentuates the impact of participating in off-farm work on family income for ethnic Korean households. This finding aligns with the findings presented in S4 Table, indicating that ethnic minority group exhibit a greater inclination towards long-distance labor engagement.

The greater positive impact of receiving welfare payments on the income of ethnic minorities, compared with that of majority ethnic group, can be attributed to the higher level of subsidies received by ethnic minorities. These subsidies encompass both general assistance provided by the Chinese government to households in need and targeted support specifically aimed at ethnic minorities and residents living in border, remote and minority areas.

Therefore, compared with the existing literature that only analyzed the direct impact of ethnicity on the income of poor households, this paper reveals the multi-influence of ethnicity on household income and its complex and diverse action paths.

### Cross-level interaction effects enhanced understanding of income outcomes among poor households

Through the application of hierarchical linear models, we confirmed that it is necessary to consider the effect of variables both at the household level and at the context level on the income of poor households for the nested structure data applied in this paper. Recognizing the mechanism and path of various factors acting on the income of poor households at different levels can help us formulate more effective targeting strategies at different levels, thereby providing an important reference for building a bridge linking the implementation of policy programs (usually implemented at the geographic area level, such as county, township, village, etc.) and their expected outcomes (usually reflected at the individual and household level) [28].

In this study, we observe that as village hollowing out intensifies at the contextual level, the decline in income associated with an increase in family size becomes more pronounced. The villages with high degree of hollowing out are mainly distributed around the county town and in the northern and eastern border areas of Wangqing County. The cost of living for households in villages around the county town is relatively high, so as the family size increases, their per capita net income decreases faster compared to other regions. The villages located in the northern and eastern border areas of the county mainly suffer from poor resources and facilities, leading to a high rate of hollowing out as a large number of farmers work outside all year round. Among these highly hollowed-out villages, larger families often have more elderly people and children with weak labor capacity, resulting in higher dependency ratios and lower per capita income. The results suggest that we should make full use of rural human capital in combination with the local situation of rural development when formulating new population structure adjustment policies to reduce poverty. However, the results also point out that adverse shocks can be buffered indirectly by encouraging people to move back to rural areas.

The increase of average slope, village size, and the ratio of ethnic minorities at the contextual level weakens the negative impact of dependency ratio on income. In our study area, the dependency ratio of poor households is primarily influenced by the number of elderly members within the household. When other conditions remain constant, the impact of the dependency ratio on household income is attenuated in villages with a higher average slope compared to those with a lower average slope. This can be attributed to the fact that villages with a lower average slope are more likely rely on agricultural cultivation, which necessitates a greater quantity and quality of labor force that may pose challenges for elderly individuals and children. Conversely, in Wangqing County’s villages characterized by a higher average slope, where the forestry resources are relatively abundant, the sale of forest products harvested beneath trees serves as a vital source of financial support for households with limited labor capacity, particularly among the elderly. Consequently, the adverse effect of the dependency ratio on income in poor households is relatively weakened in villages with a higher average slope. With the expansion of village size, the inverse relationship between dependency ratio and income of poor households tends to weaken relatively. The occurrence of this phenomenon can be attributed to the fact that larger villages are predominantly located in areas characterized by favorable resource conditions, particularly higher-quality arable land. Consequently, the elderly who no longer engage in agricultural production within these areas can also receive comparatively high income from land rent. Therefore, the adverse impact of the dependency ratio on the income of poor households diminishes with an increase in village size. In villages characterized by a higher proportion of poor ethnic minority populations, the income of poor households exhibits a comparatively slower decline as the dependency ratio increases, in contrast to villages with a lower proportion of poor ethnic minority populations. The explanation for this phenomenon lies in the results obtained from field surveys, which indicate that the average transfer income for poor ethnic minority households surpasses that of poor Han households (2,462 CNY and 2,390 CNY respectively). Transfer income serves as a primary source of livelihood for elderly poor populations in Northeast China [19]. Therefore, villages with a higher proportion of poor ethnic minorities exhibit a greater number of elderly individuals receiving transfer income compared to those with a lower proportion. As a result, an observable trend emerges wherein the adverse impact of the dependency ratio on household income diminishes as the proportion of poor ethnic minority populations in the village increases. The aforementioned findings suggest that improving the geographical conditions of rural areas and increasing the economic and social capital of villages can, to a certain extent, alleviate income poverty from high household dependency ratios.

The positive impact of off-farm work participation on the income of poor households is weakened by increases in village altitude and changes in slope. The villages located at higher altitudes are predominantly concentrated in the remote eastern regions, far removed from the town of Wangqing County. Within this specific area, under otherwise identical conditions, the rewards for engaging in off-farm work are comparatively low. Simultaneously, in the remote eastern mountainous regions of Wangqing County characterized by low urbanization levels, farmers’ engagement in off-farm activities is constrained due to their limited proficiency in non-agricultural skills [54]. Consequently, the correlation between engaging in off-farm works and income augmentation for impoverished households diminishes with increasing altitude. The villages with minimal changes in slope are predominantly situated in the southwestern region of Wangqing County, which is adjacent to the county town and offers comparatively higher remuneration for engaging in off-farm activities than other regions. Conversely, as the slope increases, there is a gradual attenuation in the positive correlation between off-farm activities and income for poor households.

The increase in both the average slope of the village and the distance from the village to the nearest river enhance the positive impact of engaging in off-farm work on the income of poor households. On the one hand, the villages with larger average slopes are predominantly located in the central and southern regions of Wangqing County, which are also in closer proximity to the county town, and also offers comparatively higher rewards for participating in off-farm activities. On the other hand, villages with larger average slopes exhibit characteristics of higher input and lower output in agricultural production. Consequently, a greater income disparity is observed between households engaged in off-farm activities and those not involved, which will also encourage more households to enhance their income through participating in off-farm activities. Under the combined effect, off-farm activities have a positive impact on the income of poor households, with this influence becoming more pronounced as the average slope of villages increases. Similarly, under constant conditions, an increase in the distance between villages and nearest rivers leads to higher irrigation costs for agricultural production and lower crop yields. Moreover, these regions exhibit heightened vulnerability to the impacts of both droughts and floods. As a result, there will also be a greater income gap between participating in non-agricultural activities and not involved. Concurrently, the limited and volatile agricultural incomes also incentivize a greater number of households to participate in off-farm works.

With the increase in the distance to the nearest township-level road, the positive impact of receiving welfare payments on the income of poor households becomes more pronounced. Villages with a long distance to the nearest township-level road are mainly located in mountainous high-altitude areas in the central and north of Wangqing County. Poor households in these remote villages often experience more disadvantages in purchasing production goods and in selling agricultural products. As one of the interviewees in our survey muttered, *“The road to my home cannot withstand the pressure of trucks loaded with corn because of its poor quality*. *After rain*, *most buyers are afraid to come here*, *so no one comes to my home to buy the corn*, *even at a low price*.*”* Therefore, households are more dependent on welfare payments, as characterized by the positive effect of welfare payments on the income of poor households increasing with the increase of the distance to the nearest township-level road.

Overall, the results of this study enhance comprehension of income outcomes among poor households and provide support for multi-level coordination in formulating poverty reduction strategies.

### The intricate interplay between household-level and environmental-level factors: Perpetuating low income in poor households

The income of households is influenced by the interplay between their inherent characteristics and external environmental factors. However, the primary determinant of low income is the inadequate livelihood capital within the household, resulting in a diminished capacity to seize opportunities, heightened levels of stress, and insufficient resilience. The huge gap between urban and rural areas has resulted in a continuous outflow of rural population, driven by the allure of external opportunities and internal decline. Left-behind families in rural regions primarily consist of women, children, and elderly individuals, leading to the erosion of grassroots organizations within these communities [55]. Consequently, the presence of an aging population, limited educational attainment, and a high prevalence of diverse ailments including severe illnesses and chronic diseases within rural households in this region result in diminished capacity of household heads to effectively manage household affairs and access economic as well as political opportunities. The combined characteristics of all family members collectively shape the level of familial stress and resilience, thereby influencing the household’s capacity to generate income. The status of family stress and its coping ability are determined by several key factors, including labor capacity as a representation of human resources, dependency ratio as an indicator of family burden, non-agricultural income reflecting income structure, and welfare payment indicating the degree of social intervention. In our study area, there is a scarcity and deterioration of human resources, an undifferentiated structure of household income, and substantial family burdens, which exert immense pressure on family development and severely constrain their adaptive capacity. Furthermore, rural families in this region exhibit a significant dependence on social interventions [19]. Therefore, the limited capacity of the household head in managing family affairs and accessing opportunities constrains the family’s ability to withstand pressure, while the high-pressure environment within the family and their insufficient resilience further undermine the household head’s abilities in managing family affairs and seizing opportunities, thus perpetuating a cycle of low income of poor households.

Finally, the underlying mechanisms driving perpetuating low income of poor households can be summarized as a complex interplay between environmental and household-level variables, mediated by industrial development and production through factors encompassing capital compensation, escape from capital dependency, and capital accumulation. Traditional farming is the major industry in rural regions, which is also the main source of income for rural households in our research area. The economic factors of low income are rooted in the persistent price gap between urban and rural products, as well as the consistently low prices of grain products. However, in regions with limited natural resources (e.g., regions with high altitudes and steep slopes that restrict agriculture-based industries), favorable conditions exist for the growth of bush collection industry. This has provided additional income opportunities for the elderly who are ill or have limited labor capacity, thus diversifying livelihoods for poor households and mitigating the negative impact of disadvantaged human capital on income. This establishes a capital compensatory mechanism between the environment and households. Meanwhile, in this process, households emancipated themselves from reliance on traditional production resources (such as arable land), thereby establishing a mechanism for escape from capital dependency. However, it is important to acknowledge that the interactive influence of specific disadvantaged capital has exacerbated the low income of poor households. For instance, marginalized geographical conditions (such as remote locations distant from markets and facilities centers) and regional natural environmental disadvantages (such as high altitude and steep slopes) impose significant constraints on families with limited resources and capabilities to engage in non-agricultural activities. Consequently, this leads to reduced wages and further intensifies the prevalence of low income. The interaction and reinforcement between geographical capital in disadvantaged areas and livelihood capital in vulnerable families create formidable barriers for these families to get rid of poverty through capital accumulation.

### Limitations and future directions

The findings in this paper enhance our knowledge of the ethnic difference of the poor households and the different effects of household-level actors, as well as the contextual effect. We recognize several limitations of this study. Due to the limited data available, this study solely focuses on examining household-level factors influencing income in poor households, as well as the ethnic disparities in these effects and contextual influences on household-level outcomes. However, it is important to acknowledge that this approach may inadvertently overlook some important information and potentially yield biased results. The results of this study are based on one case area and need further empirical testing in different areas. Therefore, future research should augment the range of sample types and expand the sample size employed to yield more robust and reliable findings. In our study, we made efforts to account for numerous variables that could have impacted the outcomes. However, there are still some unaccounted variables that may have exerted an influence on the findings. Moreover, insufficient attention has been paid to the spatial-temporal dynamics of factors at both household and contextual levels affecting income in poor households. Therefore, future research can enhance the reliability of findings and facilitate more precise assessments regarding multi-level determinants influencing income in poor households through long-term and comprehensive data collection as well as advanced statistical methodologies.

## Supporting information

S1 Table Results of the null model. (DOCX)

S2 Table Descriptive statistics and screening of variables at the household level. (DOCX)

S3 Table Results of the univariate random effect regression model. (DOCX)

S4 Table Results of the character differences in the majority group and minority group. (DOCX)

S5 Table Descriptive statistics and screening of variables at household level including household characters and interaction term of ethnicity and other characters. (DOCX)

S6 Table Household-level data. (XLSX)

S7 Table Environment-level data. (XLSX)

## References

[1] United Nations. The Sustainable Development Goals Report. New York: United Nations; 2022. https://unstats.un.org/sdgs/report/2022/

[2] Akter S, Mallick B. The poverty-vulnerability-resilience nexus: Evidence from Bangladesh. Ecological Economics. 2013; 96:114–124.

[3] Anh Tuan B, Cuong Viet N, Thu Phuong P. Poverty among ethnic minorities: The transition process, inequality and economic growth. Applied Economics. 2017; 49:3114–3128.

[4] Ngarava S, Zhou L, Ningi T, Chari MM, Mdiya L. Gender and ethnic disparities in energy poverty: The case of South Africa. Energy Policy. 2022; 161:112755.

[5] Hall GH, Harry AP. Indigenous Peoples, Poverty, and Development. England: Cambridge University Press; 2012.

[6] Jiang Y, Wang Y, Qi W, Cai B, Huang C, Liang C. Detecting multilevel poverty-causing factors of farmer households in Fugong county: A hierarchical spatial-temporal regressive mode. Agriculture (Basel). 2022; 12(11):1844.

[7] Zhou ZF, Zhu CL. Relative spatial poverty within Guizhou province, A multidimensional approach. Social Indicators. 2022; 161(1):151–170.

[8] Gustafsson B, Ding S. Temporary and persistent poverty among ethnic minorities and the majority in rural China. Review of Income and Wealth. 2009; 55:588–606.

[9] Epprecht M, Müller D, Minot N. How remote are Vietnam’s ethnic minorities? An analysis of spatial patterns of poverty and inequality. The Annals of Regional Science. 2011; 46(2):349–368.

[10] Churchill SA. Microfinance and ethnic diversity. Economic Record. 2017; 93:112–141.

[11] Barham V, Boadway R, Marchand M, Pestieau P. Education and the poverty trap. European Economic Review. 1995; 39(7):1257–1275.

[12] Bird K, Shepherd A. Livelihoods and chronic poverty in Semi-Arid Zimbabwe. World Development, 2003, 31(3):591–610.

[13] Cao MT, Xu DD, Xie FT, Liu EL, Liu SQ. The influence factors analysis of households’ poverty vulnerability in southwest ethnic areas of China based on the hierarchical linear model: A case study of Liangshan Yi autonomous prefecture. Applied Geography. 2016; 66:144–152.

[14] Chen KM, Wang TM. Determinants of poverty status in Taiwan: A multilevel approach. Social Indicators Research. 2015; 123(2):371–389.

[15] Kim R, Mohanty SK, Subramanian SV. Multilevel geographies of poverty in India. World Development. 2016; 87:349–359.

[16] Krishna A. Pathways out of and into poverty in 36 villages of Andhra Pradesh, India. World Development. 2006; 34(2):271–288.

[17] Nam SJ, Park EY. Relationship between the consumption status of households that include individuals with physical disabilities and their employment. Journal of Disability Policy Studies. 2015; 26(1):33–43.

[18] Park EY, Nam SJ. Influential factors of poverty dynamics among Korean households that include the aged with disability. Applied Research in Quality of Life. 2018; 13(2):317–331.

[19] Wang SJ, Tian JF, Wang BY, Cheng LS, Du GM. Regional characteristics and causes of rural poverty in Northeast China from the perspective of targeted poverty alleviation. Scientia Geographica Sinica. 2017; 37(10):1449–1458. [In Chinese]

[20] Zhang JP, Zuo F, Zhou YM, Zhai MX, Mei L, Fu YD, et al. Analyzing influencing factors of rural poverty in typical poverty areas of Hainan Province: A case study of Lingao County. Chinese Geographical Science. 2018; 28(6):1061–1076.

[21] Zhou W, Fang YF. Research on regional poverty trap in rural China: From the perspective of community effect. Economic Perspectives. 2012; (06):3–15. [In Chinese]

[22] Hallegatte S, Fay M, Barbier EB. Poverty and climate change: Introduction. Environment and Development Economics. 2018; 23(3):217–233.

[23] Moser C. Reducing Global poverty: The Case for Asset Accumulation. Washington, DC: Brookings Institution Press; 2008.

[24] Krishna A. For reducing poverty faster: Target reasons before people. World Development. 2007; 35(11):1947–1960.

[25] Fording RC, Berry WD. The historical impact of welfare programs on poverty: Evidence from the American states. Policy Studies Journal. 2007; 35(1):37–60.

[26] Wang YH, Liang CX, Li JC. Detecting village‐level regional development differences: A GIS and HLM method. Growth and Change. 2019; 50(1):222–246.

[27] Wang YH, Li HY. Modeling comprehensive dispersion of the administrative villages and its association with economic poverty: A case study from poverty-stricken mountainous county. China. Social Indicators Research. 2016; 133(1):1–25.

[28] Berchoux T, Hutton CW. Spatial associations between household and community livelihood capitals in rural territories: An example from the Mahanadi Delta, India. Applied Geography. 2019; 103:98–111.

[29] Kam SP, Hossain M, Bose ML, Villano LS. Spatial patterns of rural poverty and their relationship with welfare-influencing factors in Bangladesh. Food Policy. 2005; 30(5–6):551–567.

[30] Palmer-Jones R, Sen K. It is where you are that matters: The spatial determinants of rural poverty in India. Agricultural Economics. 2006; 34(3):229–242.

[31] Bloom DE, Canning D, Sevilla J. Geography and poverty traps. Journal of Economic Growth. 2003; 8(4):355–378.

[32] Gallup JL, Sachs JD. Agriculture, climate, and technology: Why are the tropics falling behind? American Journal of Agricultural Economics. 2000; 82(3):731–737.

[33] Fafchamps M, Shilpi F. Subjective welfare, isolation, and relative consumption. Journal of Development Economics. 2008; 86(1):43–60.

[34] Liang YT, Li SQ, Zeng JQ, Wu TB. Examining the impact of multidimensional accessibility on regional poverty in Laos. Applied Geography (Sevenoaks). 2022; 148:102789–. doi: 10.1016/j.apgeog.2022.102789

[35] Okwi PO, Ndeng’e G, Kristjanson P, Arunga M, Notenbaert A, Molo A, et al. Spatial determinants of poverty in rural Kenya. Proceedings of the National Academy of Sciences. 2007; 104(43):16769–16774. doi: 10.1073/pnas.0611107104 17942704 PMC2040447

[36] Omamo SW. Transport costs and smallholder cropping choices: An application to Siaya District, Kenya. American Journal of Agricultural Economics. 1998; 80(1):116–123.

[37] Bird K, Hulme D, Shepherd A, Moore K. Chronic poverty and remote rural areas. Chronic Poverty Research Centre Working Paper. 2002; (13). doi: 10.2139/ssrn.1754490

[38] Partridge MD, Rickman DS. Distance from urban agglomeration economies and rural poverty. Journal of Regional Science. 2008; 48(2):285–310.

[39] Cunguara B, Darnhofer I. Assessing the impact of improved agricultural technologies on household income in rural Mozambique. Food Policy. 2011; 36(3):378–390.

[40] Castaing MG. Education and Road Access: Empirical Evidence from Tanzania on the Complementarity Effect. Paris: International Conference on New Evidence on Poverty Traps; 2011.

[41] Carter MR, Little PD, Mogues T, Negatu W. Poverty traps and natural disasters in Ethiopia and Honduras. World Development. 2007; 35(5):835–856.

[42] Zhang LJ, Dong YM, Ha S. An analysis of spatial poverty traps in western ethnic regions. Ethno-National Studies. 2015; (01):25–35+124. [In Chinese]

[43] Gradín C. Rural Poverty and Ethnicity in China. Measurement of Poverty, Deprivation, and Economic Mobility (Research on Economic Inequality, Vol. 23). 2015; 221–247.

[44] Zainuer M. Research on the influencing factors of the poverty rate among rural elderly in southern Xinjiang—An example of survey data from five prefectures and cities. Social Security Studies. 2018; (02): 77–84.

[45] Wang BY, Tian JF, Shi X, Wang SJ. Rural poverty of Wangqing county based on HLM and GWR. Scientia Geographica Sinica. 2020; 40(03):409–418.

[46] Leeuw J. de, Meijer E. Handbook of Multilevel Analysis. 1st ed. Springer: New York; 2008.

[47] Ma L, Zhang Q, Wästfelt A, Wang SJ, Zhang Q. Understanding the spatiality of the rural poor’s livelihoods in Northeast China: Geographical context, location and urban hierarchy. Applied Geography (Sevenoaks). 2023; 152:102865–. doi: 10.1016/j.apgeog.2022.102865

[48] Luo Q, Fan XS, Gao GH, Yang HM. Spatial distribution of poverty villages and influencing factors in Qinba mountains. Economic Geography. 2016; 36(4):126–132. [In Chinese]

[49] Yang ZH, Huang QH, Li MC, Qu YB, Gao Y, Liu MF. Identification and strategy analysis of poverty alleviation traps in village area from the perspective of industry poverty alleviation: A case of Baojing County in Hunan Province. Scientia Geographica Sinica. 2018; 38(6):885–894. [In Chinese]

[50] Li S, Ding S. An empirical analysis of income inequality between a minority and the majority in urban China: The case of Ningxia Hui autonomous region. The Review of Black Political Economy. 2013; 40(3):341–355.

[51] Gustafsson B, Ding S. Why is there no income gap between the Hui Muslim minority and the Han majority in rural Ningxia, China? The China Quarterly (London). 2014; 220:968–987.

[52] Wang XF, Tian BW. Research on characteristics and influence factors of Yanbian Korean population flow. Population Journal. 2015; 37(03):78–87. [In Chinese]

[53] Li MH. Trans-boundary·identification·development: Three aspects of korean cross-border population mobility. Journal of North Minzu University. 2018; (01):38–43. [In Chinese]

[54] He RW, Fang F, Liu YW. Influence of human capital on the livelihood strategy of farming households in poor mountainous areas:A case study of Liangshan Yi Autonomous Prefecture of Sichuan, China. Progress in Geography. 2019; 38(09):1282–1293.

[55] Zhou G. The research on the structural poverty of contemporary China’s rural areas. Doctoral dissertation. Jilin University. 2018. https://kns.cnki.net/kcms2/article/abstract?v=KqXyGY4RJv2DUOifW8U258DBO7YXwJ-ozcCk3H-TONIyOX2FGJ289I-qyLg5sIkWSk8-CZMBnCztbdia2AfLF_NHVIKEUFjAUM-CoREWDRUI8sxIXNeQeX9oYN8q9WqKtBInqR3DxHUX3HaLAm1O5MO0A05Jtfmvsmpsyru4Zt0qdiDELcZHk1ZiR3_ZJGZfCXCIcH0pqoc=&uniplatform=NZKPT&language=CHS.

