# Supplementary material for: Ethnic disparities of poor households by a multilevel analysis of household and contextual effects: Evidence from a multi-ethnic county of China
Source: PLoS One. 2024 Dec 12;19(12):e0313533. doi: 10.1371/journal.pone.0313533 (PMC11637272; doi:10.1371/journal.pone.0313533)
Supplement: S1 Table — (DOCX) [file pone.0313533.s001.docx]

**S1 Table. Results of the null model**

| Fixed effects | | | | | |
| --- | --- | --- | --- | --- | --- |
|  | Parameter | Coefficient | S.E. | T-ratio | P |
| Intercept | G00 | 5899.93 | 99.23 | 59.46 | ≤ 0.001 |
| Random effects | | | | | |
|  | Parameter | S.D. | Variance Component | x^2^ | P |
| Intercept | U0 | 1259.84 | 1587194.80 | 1743.42 | ≤ 0.001 |
| Level-1 | R | 3409.17 | 11622448.75 |  |  |
