# Supplementary material for: Ethnic disparities of poor households by a multilevel analysis of household and contextual effects: Evidence from a multi-ethnic county of China
Source: PLoS One. 2024 Dec 12;19(12):e0313533. doi: 10.1371/journal.pone.0313533 (PMC11637272; doi:10.1371/journal.pone.0313533)
Supplement: S2 Table — (DOCX) [file pone.0313533.s002.docx]

**S2 Table.** **Descriptive statistics and screening of variables at the household level**

|  | Min. | Max. | Mean | S.D. | C.V. | Tol. | VIF |
| --- | --- | --- | --- | --- | --- | --- | --- |
| Income | 0 | 82 683 | 5 960 | 3 651.702 | 61.27% | - | - |
| Age | 8 | 98 | 65.12 | 12.115 | 18.60% | .350 | 2.858 |
| Gender | 0 | 1 | 0.29 | 0.453 | 156.21% | .736 | 1.359 |
| Ethnicity | 0 | 1 | 0.19 | 0.394 | 207.37% | .921 | 1.086 |
| Education | 0 | 5 | 2.34 | 0.712 | 30.43% | .782 | 1.279 |
| Disease | 0 | 2 | 0.57 | 0.605 | 106.14% | .467 | 2.139 |
| Disability | 0 | 1 | 0.42 | 0.494 | 117.62% | .380 | 2.629 |
| Labor capability | 0 | 1 | 0.28 | 0.447 | 159.64% | .464 | 2.154 |
| Family size | 1 | 6 | 1.69 | 0.736 | 43.55% | .535 | 1.871 |
| Dependency ratio | 0 | 1 | 0.70 | 0.425 | 60.71% | .423 | 2.366 |
| Student | 0 | 1 | 0.09 | 0.281 | 312.22% | .583 | 1.715 |
| Off-farm work | 0 | 1 | 0.12 | 0.325 | 270.83% | .754 | 1.327 |
| Welfare | 0 | 1 | 0.66 | 0.474 | 71.82% | .613 | 1.632 |
