# Supplementary material for: Ethnic disparities of poor households by a multilevel analysis of household and contextual effects: Evidence from a multi-ethnic county of China
Source: PLoS One. 2024 Dec 12;19(12):e0313533. doi: 10.1371/journal.pone.0313533 (PMC11637272; doi:10.1371/journal.pone.0313533)
Supplement: S3 Table — (DOCX) [file pone.0313533.s003.docx]

**S3 Table.** **Results of the univariate random effect regression model**

| Models | Regression coefficient | | | | Regression coefficient variance | | | |
| --- | --- | --- | --- | --- | --- | --- | --- | --- |
|  | Parameter | Coefficient | Standard error | P-value | parameter’ | standard error’ | variance component | |
| Y = B_0_ + B_1_*(Age) + R  B_0_ = G_00_ + U_0_  B_1_ = G_10_ + U_1_ | G_00_ | 5 896.14 | 98.59 | 0.000 | R | 3 300 | 10 890 206 | |
|  | G_10_ | −32.19 | 5.64 | 0.000 |  |  |  |  |
|  |  |  |  |  |  |  |  |  |
| Y = B_0_ + B_1_*(Gender) + R  B_0_ = G_00_ + U_0_  B_1_ = G_10_ + U_1_ | G_00_ | 5 892.85 | 98.48 | 0.000 | R | 3 384 | 11 452 147 | |
|  | G_10_ | −425.57 | 99.74 | 0.001 |  |  |  |  |
|  |  |  |  |  |  |  |  |  |
| Y = B_0_ + B_1_*(Ethnicity) + R  B_0_ = G_00_ + U_0_  B_1_ = G_10_ + U_1_ | G_00_ | 5 900.27 | 99.14 | 0.000 | R | 3 405 | 11 591 592 | |
|  | G_10_ | 17.51 | 143.81 | 0.904 |  |  |  |  |
|  |  |  |  |  |  |  |  |  |
| Y = B_0_ + B_1_*(Education) + R  B_0_ = G_00_ + U_0_  B_1_ = G_10_ + U_1_ | G_00_ | 5 889.82 | 98.43 | 0.000 | R | 3 378 | 11 410 565 | |
|  | G_10_ | 328.36 | 69.16 | 0.000 |  |  |  |  |
|  |  |  |  |  |  |  |  |  |
| Y = B_0_ + B_1_*(Disease) + R  B_0_ = G_00_ + U_0_  B_1_ = G_10_ + U_1_ | G_00_ | 5 898.99 | 98.76 | 0.000 | R | 3 389 | 11 485 208 | |
|  | G_10_ | −329.16 | 72.52 | 0.000 |  |  |  |  |
|  |  |  |  |  |  |  |  |  |
| Y = B_0_ + B_1_*(Disability) + R  B_0_ = G_00_ + U_0_  B_1_ = G_10_ + U_1_ | G_00_ | 5 888.15 | 98.52 | 0.000 | R | 3 375 | 11 489 083 | |
|  | G_10_ | −255.00 | 102.37 | 0.014 |  |  |  |  |
|  |  |  |  |  |  |  |  |  |
| Y = B_0_ + B_1_*(Labor capacity) + R  B_0_ = G_00_ + U_0_  B_1_ = G_10_ + U_1_ | G_00_ | 5 902.31 | 98.83 | 0.000 | R | 3 223 | 10 385 754 | |
|  | G_10_ | 1 611.91 | 169.35 | 0.000 |  |  |  |  |
|  |  |  |  |  |  |  |  |  |
| Y = B_0_ + B_1_*(Family size) + R  B_0_ = G_00_ + U_0_;  B_1_ = G_10_ + U_1_ | G_00_ | 5 898.55 | 99.04 | 0.000 | R | 3 394 | 11 521 005 | |
|  | G_10_ | −265.03 | 60.37 | 0.000 |  |  |  |  |
|  |  |  |  |  |  |  |  |  |
| Y = B_0_ + B_1_*(Dependency ratio) + R  B_0_ = G_00_ + U_0_  B_1_ = G_10_ + U_1_ | G_00_ | 5 894.66 | 98.58 | 0.000 | R | 3 286 | 10 800 382 | |
|  | G_10_ | −1038.62 | 156.50 | 0.000 |  |  |  |  |
|  |  |  |  |  |  |  |  |  |
| Y = B_0_ + B_1_*(Student) + R  B_0_ = G_00_ + U_0_  B_1_ = G_10_ + U_1_ | G_00_ | 5 900.97 | 99.25 | 0.000 | R | 3 405 | 11 593 028 | |
|  | G_10_ | −203.96 | 142.43 | 0.154 |  |  |  |  |
|  |  |  |  |  |  |  |  |  |
| Y = B_0_ + B_1_*(Off-farm work) + R  B_0_ = G_00_ + U_0_  B_1_ = G_10_ + U_1_ | G_00_ | 5 904.20 | 99.10 | 0.000 | R | 3 317 | 11 004 354 | |
|  | G_10_ | 1 986.70 | 218.67 | 0.000 |  |  |  |  |
|  |  |  |  |  |  |  |  |  |
| Y = B_0_ + B_1_*(Welfare) + R  B_0_ = G_00_ + U_0_  B_1_ = G_10_ + U_1_ | G_00_ | 5 892.07 | 98.32 | 0.000 | R | 3 296 | 10 865 776 | |
|  | G_10_ | −314.41 | 159.78 | 0.023 |  |  |  |  |
|  |  |  |  |  |  |  |  |  |
