# Supplementary material for: Ethnic disparities of poor households by a multilevel analysis of household and contextual effects: Evidence from a multi-ethnic county of China
Source: PLoS One. 2024 Dec 12;19(12):e0313533. doi: 10.1371/journal.pone.0313533 (PMC11637272; doi:10.1371/journal.pone.0313533)
Supplement: S4 Table — (DOCX) [file pone.0313533.s004.docx]

**S4 Table.** **Results of the character differences in the majority group and minority group**

|  |  | **Majority group (N = 8482, 80.83%)** | | |  | **Minority group (N = 2011, 19.17%)** | | | **P-value** |
| --- | --- | --- | --- | --- | --- | --- | --- | --- | --- |
| **Characters** | **Categories** | **Sample size** | **Frequency** | **Mean** |  | **Sample size** | **Frequency** | **Mean** |  |
| Age |  |  |  | 64.57 |  |  |  | 67.43 | ＜ 0.001 |
| Gender | Male | 6,156 | 72.58% |  |  | 1,304 | 64.84% |  | ＜ 0.001 |
|  | Female | 2,326 | 27.42% |  |  | 707 | 35.16% |  |  |
| Education | Illiterate or semi-illiterate | 992 | 11.69% |  |  | 79 | 3.93% |  | ＜ 0.001 |
|  | Primary school | 4,326 | 51.00% |  |  | 829 | 41.22% |  |  |
|  | Middle school | 2,919 | 34.41% |  |  | 994 | 49.43% |  |  |
|  | High school | 241 | 2.84% |  |  | 107 | 5.32% |  |  |
|  | College or above | 4 | 0.05% |  |  | 2 | 0.10% |  |  |
| Disease | None | 4,423 | 52.15% |  |  | 738 | 36.70% |  | ＜ 0.001 |
|  | Chronic disease | 3,622 | 42.70% |  |  | 1,080 | 53.70% |  |  |
|  | Serious disease | 437 | 5.15% |  |  | 193 | 9.60% |  |  |
| Disability | None | 4,788 | 56.45% |  |  | 1287 | 64.00% |  | ＜ 0.001 |
|  | Disabled | 3,694 | 43.55% |  |  | 724 | 36.00% |  |  |
| Labor capacity | No capacity | 5,997 | 70.70% |  |  | 1595 | 79.31% |  | ＜ 0.001 |
|  | Normal capacity | 2,485 | 29.30% |  |  | 416 | 20.69% |  |  |
| Family size |  |  |  | 1.72 |  |  |  | 1.53 | ＜ 0.001 |
| Dependency ratio |  |  |  | 68.51% |  |  |  | 76.51% | ＜ 0.001 |
| Student | One or more students | 794 | 9.36% |  |  | 116 | 5.77% |  | ＜ 0.001 |
|  | None | 7,688 | 90.64% |  |  | 1,895 | 94.23% |  |  |
| Off-farm work | None | 7,458 | 87.93% |  |  | 1,779 | 88.46% |  | ＜ 0.001 |
|  | Within the township | 974 | 11.48% |  |  | 205 | 10.19% |  |  |
|  | Outside the township | 50 | 0.59% |  |  | 27 | 1.34% |  |  |
| Welfare payments | None | 2,959 | 34.89% |  |  | 608 | 30.23% |  | ＜ 0.001 |
|  | Dibao or Wubao | 5,523 | 65.11% |  |  | 1403 | 69.77% |  |  |
