# Supplementary material for: Ethnic disparities of poor households by a multilevel analysis of household and contextual effects: Evidence from a multi-ethnic county of China
Source: PLoS One. 2024 Dec 12;19(12):e0313533. doi: 10.1371/journal.pone.0313533 (PMC11637272; doi:10.1371/journal.pone.0313533)
Supplement: S5 Table — (DOCX) [file pone.0313533.s005.docx]

**S4 Table.** **Descriptive statistics and screening of variables at household level including household characters and interaction term of ethnicity and other characters**

|  | Min. | Max. | Mean | S.D. | C.V. | Tol. | VIF |
| --- | --- | --- | --- | --- | --- | --- | --- |
| Income | 0 | 82 683 | 5 960 | 3 651.702 | 61.27% | - | - |
| Age | 8 | 98 | 65.12 | 12.115 | 18.60% | .320 | 3.126 |
| Gender | 0 | 1 | 0.29 | 0.453 | 156.21% | .588 | 1.701 |
| Education | 0 | 5 | 2.34 | 0.712 | 30.43% | .660 | 1.514 |
| Disease | 0 | 2 | 0.57 | 0.605 | 106.14% | .384 | 2.603 |
| Disability | 0 | 1 | 0.42 | 0.494 | 117.62% | .309 | 3.238 |
| Labor capacity | 0 | 1 | 0.28 | 0.447 | 159.64% | .390 | 2.567 |
| Family size | 1 | 6 | 1.69 | 0.736 | 43.55% | .463 | 2.158 |
| Dependency ratio | 0 | 1 | 0.70 | 0.425 | 60.71% | .350 | 2.860 |
| Off-farm work | 0 | 1 | 0.12 | 0.325 | 270.83% | .628 | 1.592 |
| Welfare | 0 | 1 | 0.66 | 0.474 | 71.82% | .472 | 2.117 |
| Ethnicity*Age | 0 | 97 | 12.92 | 27.023 | 209.11% | .025 | 39.466 |
| Ethnicity*Gender | 0 | 1 | 0.07 | 0.251 | 371.94% | .431 | 2.320 |
| Ethnicity*Education | 0 | 5 | 0.49 | 1.050 | 213.90% | .076 | 13.206 |
| Ethnicity*Disease | 0 | 2 | 0.14 | 0.396 | 283.62% | .170 | 5.881 |
| Ethnicity*Disability | 0 | 1 | 0.07 | 0.253 | 367.33% | .227 | 4.407 |
| Ethnicity*Labor capacity | 0 | 1 | 0.04 | 0.195 | 492.75% | .381 | 2.626 |
| Ethnicity*Family size | 0 | 6 | 0.29 | 0.671 | 229.03% | .139 | 7.206 |
| Ethnicity*Dependency ratio | 0 | 1 | 0.15 | 0.348 | 237.23% | .099 | 10.137 |
| Ethnicity*Off-farm work | 0 | 1 | 0.02 | 0.147 | 665.38% | .491 | 2.038 |
| Ethnicity*Welfare | 0 | 1 | 0.13 | 0.340 | 254.56% | .209 | 4.793 |
